# Values related to professionalism in dental education at the University of Chile: Student and faculty perceptions

**DOI:** 10.1111/eje.12419

**Published:** 2019-02-19

**Authors:** Marcela Alcota, José M. Manríquez, Marco Cornejo‐Ovalle, Juan C. Salinas, Victor M. Catano, Pilar Ruiz de Gauna, Fermín E. González

**Affiliations:** ^1^ Department of Conservative Dentistry, Faculty of Dentistry University of Chile Santiago Chile; ^2^ Institute for Research in Dental Sciences, Faculty of Dentistry University of Chile Santiago Chile; ^3^ Department of Prosthetics, Faculty of Dentistry University of Chile Santiago Chile; ^4^ Department of Psychology Saint Mary’s University Halifax Nova Scotia Canada; ^5^ Faculty of Education University of Basque Country, UPV/EHU Bilbao Spain

**Keywords:** dental education, professionalism, values

## Abstract

**Objective:**

It is recognised that professionalism should play a central role in dental education. However, its implementation into the curricula of dental schools is still limited. Our objective was to identify the main values related to professionalism based on the perceptions of students and faculty members from the Faculty of Dentistry, University of Chile.

**Methods:**

A Dental Values Survey was validated and culturally adapted in order to guarantee the greatest possible internal validity. The adapted survey was administered to students and faculty members (416 and 225, respectively). The final survey contained 64 items rated on a Likert scale of 1‐5. Each item was categorised according to five dimensions: Altruism, Consciousness, Personal Satisfaction, Quality of Life and Professional Status. The values were compared between faculty and students and among students at different courses. A values scale was constructed by selecting the five items with the highest average score for each dimension.

**Results:**

Survey respondents composed 34.32% of the universe, of which 50.46% were faculty and 49.54% were students. Values associated with Altruism, Consciousness and Professional Status, were the highest rated by students and faculty. Values associated with Personal Satisfaction and Quality of Life received the lowest scores for both groups.

**Conclusions:**

To provide the best possible attention to patients (Consciousness), and that patients have access to affordable dental care (Altruism), are the values at the top of our scale. On the other hand, to maintain financial stability and to be well paid (Quality of Life) were the less considered.

## 1 INTRODUCTION

There are several definitions of professionalism in the literature such as the stated by Masella who considers that is the display of high intellectual, technical, and moral qualities and abilities, in service to patients and community^1^ or the definition proposed by Trathen and Gallagher which is an amended form from the Royal College of Physicians and states that dental professionalism implies a set of values, behaviours and relationships that underpins the trust the public has in dentists.^2^ However, all of them are related to the qualities, values and conducts that must be shown by a person in the specific context of a profession.^1^, ^3^

A number of professional associations, dental schools and researchers in dentistry consider training on professionalism of great relevance for the future of dentistry. Indeed, many hold the view that dental schools are responsible for developing academic lesson plans that incorporate both specific technical skills and transversal competencies to ensure the development of certain attitudes, values and ethical behaviours among students.^1^, ^4^, ^5^, ^6^, ^7^, ^8^ Existing evidence in dental education suggests that the professional attitudes taught to students in dental programmes will greatly determine the attitudes with which students will finally exercise their profession as a dentist.^9^ Therefore, early instruction on professional attitudes and on related educational values is of notable importance, where dental students, and future dentists, can effectively be taught the level of professionalism required to meet ethical standards in dentistry. Ultimately, the application of this approach will result in better dental care for patients.^10^, ^11^, ^12^, ^13^

In addition, the climates and educational contexts created within the faculties or schools of dentistry, derived from the challenge of patient clinical care, clinical programmes to be followed, and teaching performances, often promote individualism, competence and stress in the students, all factors to the detriment of learning the values of professionalism during dental training.^8^, ^14^, ^15^, ^16^ Considering this, the efforts made by faculty members to create an academically inclusive and professional climate are also relevant for student education. Specifically, having an integrated learning environment constantly reinforces positive aspects from the first day of a student's education. Furthermore, having such a climate would leave no doubt for faculty members, administrative staff and students as to what constitutes acceptable and unacceptable behaviour, whether in clinical or academic environments.^17^

In addition to specific professional competencies, efficient professional development of a dentist should contemplate transversal or generic competencies, which are mainly related to personal development, attitudes and professional behaviour. An important aspect regarding generic competences is that they involve ethics and values as part of a competent behaviour, and therefore, the acquisition and development of these competencies cannot be left to chance or in the hands of the student. Instead, it is the role of educational institutions to instil these values and ensure they are reflected by the attitudes and actions of the faculty.^18^ It has been described that in order to properly integrate the values of professionalism during professional training, it is relevant, as a first step, to identify what the core values important to encourage for the institution are, being surveys useful tools that could help in this first step.^19^ In a dental context, one of the few instruments that allows this first approach to the values of professionalism is the Dental Values Survey, developed and applied in Canada by Langille et al,^20^ which helps to establish a Dental Values Scale. However, there is no Spanish version of this tool to apply in dental schools from Spanish speaking countries.

The objective of the present study was to identify the values closely related to professionalism from the standpoints of students and faculty members from the Faculty of Dentistry at University of Chile through a validated survey administered to dental students and faculty members of the institution.

## 2 MATERIALS AND METHODS

The protocols for research and survey administration were evaluated and approved by the Scientific Ethical Committee of the Central Metropolitan Health Service of Chile's Ministry of Health (certificate number 23/15).

### 2.1 Cultural adaptation and validation of a Dental Values Survey

The survey developed by Langille et al,^20^ which created a values scale associated with professional conduct at a dental school in Canada, was culturally adapted and validated for use in the present study. Indeed, it has been demonstrated that preferences regarding professional values are strongly defined by social and cultural factors that are characteristic in a specific population.^21^ Cultural adaptation and validation of the survey for the Chilean context included the following stages: (a) initial translation of the original survey following standardised procedures to ensure semantic equivalence between the original and translated content; then, the back translation of this first version into the original language; (b) survey validation by a panel of five experts, all of them with an extensive training and experience in dental education; and (c) exploratory pilot study in which the agreed‐upon version of the survey was randomly administered (N = 100) to the target population, including faculty and students of the Dental Program of the Faculty of Dentistry at the University of Chile.

Once the pilot study was ended, the survey results were statistically analysed to determine internal consistency. The survey was considered sufficiently reliable, returning a Cronbach's *α* of 0.94 for the entire survey. Each survey item was grouped according to the following five dimensions: Altruism, Personal Satisfaction, Quality of Life, Consciousness and Professional Status. Langille et al constructed the operational definitions and associated values of these dimensions based on definitions provided by the American Dental Education Association (ADEA) and on the ethical principles and professional codes of conduct of the American Dental Association (ADA).^6^, ^7^, ^20^ Following this, a simple exploratory factor analysis and a principal components analysis were performed by dimension for each item using the STATA 12 software (StataCorp, College Station, TX, USA). This analysis removed items insufficiently associated with the dimensions of the model, resulting in a final survey comprised of 64 items. At the end of the survey, general demographic questions were asked, including age, sex and level of specialisation (faculty) or programme year (students). An open space was also provided for additional comments.

### 2.2 Procedure for administering the Dental Values Survey

To deliver the survey to the target population, the final adapted and culturally validated survey was sent by email to the faculty members and undergraduate students from the Faculty of Dentistry at the University of Chile (N = 641; 225 faculty and 416 students), through an online platform (Survey Monkey, San Mateo, CA, USA). For faculty members, the only exclusion criterion applied was to have a profession different from dentist (biochemist, biologist, speech therapist, etc). The emails invited faculty and student participation in the survey, explained the objectives of the survey, guaranteed confidentiality, clearly expressed that participation was voluntary, and that participants could terminate involvement at any moment during the survey. Before beginning the survey, each participant had to click to provide informed consent, and only then, the survey material was shown. The survey was open‐access and available for four weeks, with two reminders sent to the target population during this period.

### 2.3 Information analysis

Descriptive analyses were performed for the study group, specifically of frequencies and percentages in regards to sex and level of specialisation/programme year. The averages obtained per items within each dimension were compared between faculty and students and between students according to programme year. Significant differences were established by Student's *t* test when data were normally distributed and the Mann‐Whitney U test for non‐parametric data. Additionally, the opinions/comments of participants were assessed by inductive qualitative analysis using the N‐Vivo program (QSR International, Melbourne, VIC, Australia).

## 3 RESULTS

Faculty and students (N = 220) from the Faculty of Dentistry at the University of Chile, representing 34.32% of the available universe, completed the survey. Of these respondents, 50.46% were faculty and 49.54% were students. Table 1 presents the general characteristics of participants: age, sex, number of students and faculty members, and level of training.

**Table 1:** General characteristics of survey participants

Variables		Average	Range
Age (years)	Students	23.2	18‐28
	Instructors	38.0	26‐71
		**N**	**%**
Sex	Male	83	43.5
	Female	108	56.5
Level of training	Students	109	49.5
	Dentists	111	50.5
	General	41	36.9
	Specialised	70	63.1

When comparing the average scores for items from each of the five survey dimensions, Quality of Life, which was primarily related to obtaining a good and comfortable life, as well as achieving financial stability and security, was one of the lowest rated dimensions and did not show significant differences between faculty and students (4.04 ± 0.3 vs 4.01 ± 0.36, respectively; *P* > 0.05; Table 2). The dimension Personal Satisfaction, with items referring to having a harmonious and enjoyable life outside of work/study and to being happy, was the second lowest rated dimension in both groups and significantly lower for students (4.26 ± 0.22 vs 4.22 ± 0.39, respectively; *P* < 0.01; Table 2).

**Table 2:** Comparison of dimension averages between students and instructors. Average scores are presented on a Likert Scale of 1‐5

Dimension (Cronbach's *α*)	Average faculty (SD)	Average students (SD)
Altruism (*α* = 0.93)	4.42 (0.27)	4.41 (0.13)
Personal Satisfaction (*α* = 0.93)	4.26 (0.22)	4.22* (0.39)
Consciousness (*α* = 0.92)	4.57 (0.21)	4.28* (0.37)
Quality of Life (*α* = 0.89)	4.04 (0.30)	4.01 (0.36)
Professional Status (*α* = 0.74)	4.32 (0.31)	4.24 (0.34)

SD, standard deviation.

* *P* < 0.01 significantly less in students.

In contrast, Altruism, defined as behaving with compassion, demonstrating kindness and understanding others, did not present significant differences between instructors and students, in addition to being the highest rated of the evaluated professional dimensions (4.42 ± 0.27 vs 4.41 ± 0.13, respectively; *P* > 0.05; Table 2).

When comparing and analysing the results of the different values associated with each dimension among students from different course years, those related to the different dimensions evidenced an abrupt drop in the fourth year, with significant differences against most of the other years. This drop was considerably recovered when students entered the sixth (ie final) year of study, with better scores in all the dimensions in this year and three of them significantly higher compared with the fourth year (Altruism 4.61 ± 0.29 vs 4.45 ± 0.26; Personal Satisfaction 4.55 ± 0.21 vs 3.80 ± 0.36; Quality of Life 4.00 ± 0.29 vs 3.46 ± 0.26; Table 3).

**Table 3:** Comparison of dimension averages between students according to programme year. Average scores are presented on a Likert Scale of 1‐5

Dimension (Cronbach's *α*)	Student averages by programme year (SD)			
	1st‐3rd year (basic courses)	4th year (start attending patients)	5th year	6th year
Altruism (*α* = 0.93)	4.4* (0.26)	4.0* (0.32)	4.2 (0.23)	4.6* (0.29)
Personal satisfaction (*α* = 0.93)	4.4† (0.17)	3.8† (0.36)	4.2† (0.31)	4.5† (0.21)
Consciousness (*α* = 0.92)	4.5‡ (0.23)	4.2‡ (0.27)	4.5‡ (0.26)	4.7 (0.21)
Quality of life (*α* = 0.89)	3.9§ (0.32)	3.4§ (0.26)	3.6 (0.17)	4.0§ (0.34)
Professional status (*α* = 0.74)	4.3 (0.26)	3.8 (0.46)	4.1 (0.33)	4.3 (0.29)

SD, standard deviation.

*,†,‡,§ *P* < 0.05 significantly lower scores for fourth‐year students.

The optional opinions of students and faculty, assessed via inductive qualitative analysis, were grouped into two categories: perception of the profession and aspects related to training. Subcategories were assigned to each of these two categories, which, in addition to all the responses, are summarised in Table 4.

**Table 4:** Comparison of open opinions between students and faculty members (number of opinions)

Category	Subcategory	Faculty opinions	student opinions
Perception of the profession	Vocation	This profession requires a vocation for service^3^	A vocation to help others^3^
		Important in society^1^	
	Work/Life Balance	There is no time^2^	Preoccupation of not having time for family^1^
	Financial Stability	Poor payment, need various jobs^3^	Insecurity in regard to finding a job^2^
Aspects related to training	Professionalism Training	Important to provide education on professional values and social commitment^3^	Integrate multidisciplinary teams^2^
			The programme does not provide training for this aspect^3^
			Revalue undergraduate education, oriented to form good general and dentists with integrity^2^
	Stress		Emotionally draining and stressful degree programme^3^

The category perception of the profession is related to all the opinions, from students and faculty members, regarding their experiences when performing the profession. The comments in this category were related, on one hand, to the fact that they think that to study dentistry you need to have the real vocation to help others and, on the other hand, to the fact that when practising dentistry, it is difficult to reconcile your time with family life, and that there is work instability and low employability once graduated. Representative quotes of this category are shown in Box 1.

Box 1 **Representative quotes of teachers and students related to the category perception of the profession**

1 “Although I love my profession, the current conditions are creating an environment of general disillusionment due to an oversupply of dentistry schools, poor working conditions, unequal access of the population to dental health, bad public policies, etc…today, one has to work by volume to have economic stability, which finally prejudices the goal of providing health to others and having health oneself.”—Faculty

“The most complicated aspect for me has been to balance my professional and family/personal lives, with very little time left for the latter.”—Faculty

“I feel unsure about my opportunities for work and economic stability once I graduate, which gives me a high degree of anxiety and dissatisfaction, especially after having studied dentistry for six years, in addition to an associate's degree.”—Student

“The most important thing to have as a health professional, no matter the field, is to have a calling to help others, to want to better the life of someone and to solve their ailments.”—Student

The category professional formation was related to the students’ and faculty members’ opinions regarding the topics about professionalism that should be taught and/or encouraged to students during the dental programme. Representative quotes of this category are shown in Box 2.

Box 2 **Representative quotes of faculty and students related to the category professional formation**

1 “The mission of dental education should be to train future dentists with strong dedications to the society and to decreasing indices of morbidity, increasing universal access to dental treatments.”—Faculty

“I consider it important to work on professionalism while training students.”—Faculty

“I am happy, but training to be a dentist is a process that is decreasing my happiness. There is constant stress and little social life.”—Student

“In the survey, I responded ‘in disagreement’ or ‘very in disagreement’ to questions associated with my current reality as a dental student, eg items related to my quality of life while I am studying at the faculty. As students, we are generally submitted to destitute conditions!”—Student

A scale constructed based on the values most closely related to professionalism, according to the perceptions of students and faculty from the Faculty of Dentistry at the University of Chile, revealed that the values of providing the best possible attention to patients (Consciousness), having access to dental treatment (Altruism), the importance of continued professional development (Professional Status) and being honest (Consciousness) led the scale, with average scores between 4.87 and 4.75. On the other hand, while financial stability and economic stability were important for the survey participants, all of the items linked to the Quality of Life dimension were placed lowest on the scale, with average scores between 3.84 and 3.68 (Table 5).

**Table 5:** Values scale constructed according to the opinions of dental students and faculty members from the Faculty of Dentistry at the University of Chile

Value associated with dental professionalism	Average score (SD)
I provide the best possible attention to my patients (Consciousness)	4.8 (0.47)
All of my patients have access to affordable dental care (Altruism)	4.7 (0.53)
I consider continued professional development important (Professional Status)	4.7 (0.50)
I am honest (Consciousness)	4.75 (0.65)
I conscientiously perform my job (Consciousness)	4.7 (0.58)
I act ethically (Consciousness)	4.7 (0.60)
I am trustworthy (Consciousness)	4.6 (0.64)
I respect others (Altruism)	4.6 (0.60)
I value other people (Altruism)	4.6 (0.57)
I show empathy with my patients (Altruism)	4.6 (0.62)
I am friendly with others (Altruism)	4.5 (0.62)
I respect myself (Personal Satisfaction)	4.4 (0.80)
My job is related to the medical field (Professional Status)	4.4 (0.76)
I have expectations of career progress (Professional Status)	4.4 (0.75)
I am happy with my life (Personal Satisfaction)	4.3 (0.76)
I am comfortable with who I am (Personal Satisfaction)	4.3 (0.84)
I achieve personal satisfaction (Personal Satisfaction)	4.3 (0.76)
My life is enjoyable (Personal Satisfaction)	4.2 (0.76)
I have a good quality of life (Quality of Life)	4.0 (0.89)
I have excellent technical abilities (Professional Status)	4.0 (0.77)
I am able to balance my work and personal lives (Personal Satisfaction)	4.0 (0.93)
I have exceptional clinical training (Professional Status)	4.0 (0.89)
I am economically stable (Quality of Life)	3.8 (0.94)
I maintain financial stability (Quality of Life)	3.7 (1.01)
I am paid well (Quality of Life)	3.6 (1.05)

## 4 DISCUSSION

The present study validated and administered a Dental Values Survey to faculty members and students from the Faculty of Dentistry at the University of Chile in order to identify the most important professionalism‐associated values. Different works have demonstrated the relevance of this aspect in medical and dental education, describing that in order to introduce professionalisms into the curriculum, it is previously necessary to establish an own definition of professionalism and its values, reaching a consensus between students and faculty members’ opinions (or even other members of the academic community). Once the relevant professional values for a specific institution and its context identified, their introduction into the curriculum through vertical integration in a spiral curriculum and using proper methodological and evaluative tools is mandatory.^19^, ^22^

In this study, the dimension Quality of Life was scored the lowest, without significant differences between faculty and students. This could be principally due to the current market situation for the dental profession in Chile. Specifically, the lack of regulation on the quantity of dental programmes being offered has resulted in high enrolments and an inflated number of dentistry graduates in recent years, and the labour market has not been able to absorb these numbers, creating a complex job market for dentists.^23^ This situation has been mostly prevalent in the first generations of dentists that are unemployed or underemployed, principally by private clinics or “mega”‐health providers. Many are paid a percentage of services rendered, which varies depending on the number of patients. This is in addition to forgoing many traditional benefits, such as a work contract, health insurance and workers’ compensation.^24^ Particularly in regard to students, other factors that may contribute to the perceived low quality of life include a demanding educational programme that leaves little free time and insecurity about real job prospects upon graduating, resulting in high degrees of frustration and anxiety according to student opinions (Box 1).

When we compare our results with those obtained by Langille et al, we observed that the dimensions Quality of Life and Personal Satisfaction were lower in our reality. This can be explained, at least in part, by the Chilean context and environment for dental professionals in comparison with the context in Canada. Currently, Chile has 34 dental schools with a total of 22 859 dentists^25^ for a population of 17 million people, with a dentist to population ratio of 1:760. In Canada, there are just 10 dental schools^26^ for a country with 37 million people and with a dentist to population ratio of 1:1622.^27^ Related to this, the recommended ratio from the Organisation for Economic Co‐operation and Development (OECD) is 1:1785 which means that, in total, Chile has approximately a 230% oversupply of professionals. However, if we analyse the distribution of dentists between public and private sectors, public sector, with approximately 4500 dentists,^25^ provides care to about 80% of the population (~13.6 million people) and, therefore, has an approximately 100% deficit of dentists with an important overload of work for these professionals. On the other hand, if we consider that private sector gives dental treatment to the remaining 20% of the population (~3.4 million people), it has about a 1000% oversupply (1:185 dentist to population ratio), which means a highly competitive scenario, specially for future dentists. In this context, this situation is very likely to have a negative influence in students’ and dentists’ perception regarding their Quality of Life and Personal Satisfaction. Moreover, in Canada there is the National Dental Examining Board, which ensures a standard after graduation for all dentists in the country, and in Chile, just 16 out of 34 dental schools have a proper accreditation, which ensures some quality standard of their graduates.^28^ This situation may reflect a more unstable situation for dentists in Chile than in Canada, which could be responsible for the lower score for these dimensions.

The second lowest rated dimension was Personal Satisfaction, with student ratings significantly lower than those of faculty. These results contrast with that reported by Langille et al,^20^ a study in which students reported significantly higher personal satisfaction than faculty. This difference could be explained by the stress and emotional exhaustion that dental students undergo during training, where deadlines must be met, participation in a number of clinical modules in different sub‐disciplines is required, and failure means retaking courses, thus delaying the entire programme and graduation.^29^, ^30^, ^31^ The stress of students could also increase as a result of variables or situations out of their control, such as obtaining patients to perform specific treatments required to pass each clinical module, getting the laboratory to properly perform work and deliver on‐time and having patients attending appointments, with punctuality and following treatment indications, among other factors. Similarly, many of the variables that determine the successful completion of dental training are not only related to the technical abilities of students, which does stress a percentage of students, but also with the ability to communicate and establish a good relationship with patients, requiring skills many times inadequately taught within the programme curriculum.^32^, ^33^, ^34^, ^35^ In addition, this difference between students and faculty members can be partially explained by the fact that being a professor can give an additional professional and social recognition, compared with a college student. This could be especially relevant in our university, the oldest in Chile, where many faculty members have a strong commitment and are proud to be part of the institution. When comparing these situations with the Canadian context, the differences can be explained by the fact that many dental schools in Canada have developed and applied more innovative teaching methods that better prepare students for future professional challenges. For instance, some dental schools use standardised patients for training in professionalism, communication with the patient and the critical thinking of the students. This is additionally complemented with jurisprudence courses. Altogether, these approaches could give more opportunities to students in order to develop communication skills and more experience in making clinical decisions when treating patients.^17^

Considering this context, the lower ratings by students in the present study in both Quality of Life and Personal Satisfaction could be explained by the combination of variables related to the degree programme itself as well as to the lack of job security in relation to expectations due to the current state of dentistry in Chile (Box 2).^24^ The impact of these variables becomes more acute when adding that an important percentage of the students enrolled in the Dental Program at the University of Chile are the first generation in their families to attend higher education. This likely adds familial expectations for better opportunities and social mobility, which may increase stress, and is something that is currently not secure for programme graduates.

Although higher than students, the low personal satisfaction of faculty members, as mentioned in their comments during the survey, is probably due to the little free time available for personal social activities (Box 1). This lack of time is a result of teaching duties and the necessity to have various employers for clinical practice, once again as a consequence of the aforementioned market saturation of dentists.^24^ Furthermore, working as a faculty member at the Faculty of Dentistry does not only mean supervising the clinical work of students, which consumes most paid hours, but also implies unpaid overtime to complete tasks such as class and seminar preparation and grading quizzes/tests. This work situation undoubtedly contributes to a deteriorated perception of the work‐life balance and little time for personal and family activities (Box 1).

In the dimension Consciousness, encompassing professional values of acting ethically and honestly, it was interesting to find that although it was the second highest rated dimension, indicating its value of importance, students rated this dimension significantly lower than faculty. This is in line with that previously reported by Langille et al^20^ and might be explained, in part, by the dishonest actions that students have admitted to taking to prevent programme failure.^14^, ^36^, ^37^ Moreover, it is probable that the high enrolment costs and debt associated with financing higher education in Chile further promote unethical behaviour. The present results could also be associated with previous studies, which indicate that existing environments in dentistry faculties favour individualistic and competitive behaviour over personal development.^37^, ^38^, ^39^ Due to the variety of interactions that can occur when students are attending patients, it is reasonable to assume that the hidden curriculum (teachings transmitted to students that does not explicitly appear in the formal curriculum) becomes more active in a clinical context, where the supervising instructor should act as a model for students to follow in regard to attitude and behaviour. In this context, instructors should also act as moral models and receive training on the educational system.^12^

The dimension Professional Status did not present significant differences between students and instructors, and this was similar to the previously reported in Canada. However, it is worth mentioning that the two most important items for both groups were continued professional development and that the profession is related to the medical environment. These aspects are fundamental in the educational environment of today. This is especially true in health professions given the paradigm shift in skill formation, where it is necessary to train dentists able to critically self‐assess and continuously update their knowledge.^40^

The values associated with Altruism obtained the highest averaged values, without significant differences between instructors and students. These results indicate that those who study dentistry at the University of Chile, in large part, do so as a vocation for helping people and that this premise is maintained not only while studying, but also after graduation. This result becomes relevant if institutions assume student education not only from a technical standpoint, which is undoubtedly the first moral responsibility, but also from a standpoint of training professionals that will contribute to a better, more humane and more just society.^41^, ^42^ In Canada, although the values for this dimension were also high, the highest dimension was Conscientiousness. In our opinion, this difference is due, at least in part, to social and health inequities, as well as to difficulties to access dental care for the most vulnerable population in Chile. In this context, students at the University of Chile, the main public university in the country, are probably more prone to face these inequities (social consciousness), especially if we consider that many of them have suffered those situations and are the first generation of professionals in their families. As highlighted by Dharamsi et al, the value of social responsibility mainly comes from being aware of social inequalities and is strongly linked to social consciousness, which comes together with an ethic of care and trust beyond individualism and private interests.^43^, ^44^, ^45^

Finally, one of the most important results obtained when comparing the different dimensions between students from different course years was that values associated with the dimensions Quality of Life, Personal Satisfaction, Altruism and Consciousness abruptly fell in the fourth year of the programme, with significant differences as compared to other years. These values considerably increased when students entered the sixth, and final, year of the programme. This is in contrast with that reported by Langille et al who described that values associated with the different dimensions were consistently decreasing during the years, being specially lower in the last year when compared with the first one. They described that this situation may represent a “relaxation” effect in that senior students know the realistic amount of work needed to complete the programme and, therefore, they would need some reinforcement to prevent conscientiousness from slipping further and to give the fourth‐year students a more realistic expectation of the level of conscientiousness needed for success in dental practice.^20^ In the present study, fourth‐year students must attend real patients for the first time, which could contribute to emotional burnout, pressure and stress, as previously analysed. In contrast, years one to three of the programme are focused on basic science and preclinical practices. In the sixth year, students finalise their school‐based training and enter residency programmes at hospitals and rural/urban primary care centres. While faced with new environments and challenges, during residencies, students are freed from the pressures of completing specific clinical modules and obtaining patients. It is also possible that by fully executing their profession, sixth‐year students are “reenchanted” by their selected major, which continues to hold high social recognition.

## 5 CONCLUSIONS

The closest professionalism‐associated values for faculty members and students from the Faculty of Dentistry at the University of Chile were Altruism, Consciousness and Professional Status, being the highest scores (with punctuations between 4.87 and 4.73): to provide the best possible attention to my patients (Consciousness), to have access to affordable dental care (Altruism), the relevance of continuing professional formation (Professional Status), being honest (Consciousness), I conscientiously perform my job (Consciousness) and I act ethically (Consciousness).

On the other hand, the weakest values for students and faculty members are those related to Personal Satisfaction and Quality of Life, being the items I maintain financial stability and I am paid well, both related to Quality of Life, with the lowest scores (3.75 and 3.68, respectively).

There are decreased scores for values associated with professionalism in fourth‐year students in the dimensions of Altruism, Consciousness and Quality of Life, as a consequence of the pressure and stress that students are subjected to when beginning the clinical attention of patients, which requires meeting a number of compulsory programme and course requirements to pass. However, these dimensions are recovered in the last year of training.

## 6 STUDY LIMITATIONS

In our opinion, the main limitation of this study is related to the instrument applied (Dental Values Survey) which, as a quantitative technique for data collection, does not go further regarding the description of the identified values. However, the application of this instrument constitutes the first approach to identify the most important professional values for students and faculty members during dental training and further studies are needed to address this issue.

An additional limitation is related to the fact that the survey was applied in a faculty of dentistry. This was not only a methodological decision but also a conceptual one since we were mainly interested in identifying, from a critical social perspective of understanding education, our institutional situation regarding this topic, in order to further transform and enhance their development in our students. We also believe that our institution may reflect and influence an important part of the dental reality in our country. Indeed, the Faculty of Dentistry at the University of Chile is the oldest (with more than 100 years of history) and the most important in Chile, being the only Chilean dental school in the prestigious Academic Ranking of World Universities 2018 (ranked 101‐150).

## 7 PERSPECTIVES

Currently, the Faculty of Dentistry at the University of Chile is in the fifth year of innovating its curriculum, with specific initiatives to have students practice in integrated clinics, thus bringing the student closer to real clinical situations from earlier stages of training. Gaining clinical competencies by providing comprehensive patient care and not by simply fulfilling individual tasks could be an opportunity to significantly decrease the stress of students, in addition to strengthening their sense of responsibility and empathy with patients. This, in turn, will prevent seeing the patient as an object or programme to complete, but rather as a person who needs help to resolve their oral health problems and recover their general well‐being. This earlier in‐clinic practice will also give students the opportunity to work the values of professionalism.

In relation to the dental profession, there is an exaggerated number of Dental Schools in Chile, many of which are of doubtful quality. Due to this, we believe it is of vital importance to address and review the different existing programmes, with the obligatory accreditation of individual dentistry programmes being a fundamental and urgent basic requirement to ensure that graduates are properly trained. Stricter institution and programme requirements will prevent high graduate numbers and, consequently, decrease the future unemployment or unstable employment of new professionals. In a later stage, specific and transversal competency levels could be required from graduates via a Standardized National Dentistry Exam, the application of which has an extensive track record worldwide.^46^, ^47^ Passing this exam should be an indispensable requirement for dentists in Chile, in both public and private sectors. These steps would grant greater confidence in the quality of dental programmes as well as for the graduates from these programmes, changes that would significantly improve the quality of life and oral health of the Chilean population.

## CONFLICT OF INTEREST

The authors declare that there is no conflict of interest regarding the publication of this article.
